# An epidemiological trend analysis of oral cancer in Korea from 2001 to 2021

**DOI:** 10.1186/s12903-025-06351-1

**Published:** 2025-07-01

**Authors:** Hye-Sun Shin, Junho Choi, Yuyi Park, Uiseop Shin, Sangmyeong Kim, Dong-Hun Han

**Affiliations:** 1 Department of Dental Hygiene, Dongnam Health University, Suwon-Si, Gyeonggi-Do 16328 Republic of Korea; 2 Department of Electrical and Computer Engineering, Seoul National University, Seoul, 08826 Republic of Korea; 3 Department of Preventive and Social Dentistry School of Dentistry, Seoul National University, 1, Gwanak-ro, Guanak-gu, Seoul, 08826 Republic of Korea; 4 Dental Research Institute, Seoul National University, Seoul, 08826 Republic of Korea

**Keywords:** Oral cancer, Annual percentage change, Incidence, Epidemiology

## Abstract

**Background:**

Although recent global studies have highlighted shifting patterns in oropharyngeal cancer, Korea lacks comprehensive epidemiological data on oral cancer, including detailed prevalence by anatomical subsites, age, and sex. This study aims to analyze long-term trends in oral cancer incidence and mortality in Korea, with particular attention to disease spectrum across subsites.

**Methods:**

Patient data from individuals diagnosed between 2001 and 2021 were obtained from Cancer Registration Statistics by the Ministry of Health and Welfare, while cause-of-death statistics from Statistics Korea provided information on deaths due to malignant neoplasms of the lip, oral cavity, and pharynx. Age-standardized incidence and mortality rates were calculated using the direct method, with the 2000 Korean mid-year population as the standard. Temporal trends were assessed using joinpoint regression analysis to estimate annual percentage changes (APCs) and to identify significant shifts in incidence and mortality across sex, age group, and subsites.

**Results:**

The overall trend in oral cancer incidence among males was modest (APC: 0.30), with the most notable increase observed in those aged 45 or younger (APC: 2.13). In females, oral cancer incidence showed a significant increase (APC: 2.19), with the highest rise occurring in those aged 45 or younger (APC: 3.20). Subsite-specific trends showed that in males, the greatest increases were in cancers of the tonsils (APC: 3.11), salivary glands (APC: 2.01), and tongue (APC: 1.95), whereas in females, the tongue (APC: 3.97), tonsils (APC: 3.85), and salivary glands (APC: 3.09) showed the highest increases.

**Conclusions:**

These findings demonstrate distinct epidemiological shifts in oral cancer in Korea over the past two decades, particularly by age, sex, and anatomical subsite. While incidence increased notably among younger females, mortality declined in males, suggesting improved survival outcomes. These results underscore the importance of continuous monitoring and subsite-specific prevention strategies tailored to evolving demographic and clinical patterns.

**Supplementary Information:**

The online version contains supplementary material available at 10.1186/s12903-025-06351-1.

## Background

Oral cancer, including lip, oral cavity, and pharyngeal cancers, poses a significant global health burden. According to the Global Burden of Diseases (GBD) Study 2019, an estimated 370,000 new cases of lip and oral cavity cancer and 167,000 cases of other pharyngeal cancer occurred worldwide in 2019 [1]. Additionally, there were approximately 199,000 deaths attributed to lip and oral cavity cancer and 114,000 deaths due to other pharyngeal cancers globally [1]. This contributed to the loss of 5.5 million and 3.2 million disability-adjusted life years [1]. According to the 2021 National Cancer Registry Statistics of Korea, oral cancer accounted for approximately 1.6% of all cancer cases. In men, oral cancer ranked as the 10th most common cancer [2]. Despite the decline in smoking rates due to effective tobacco control policies, the incidence of oral cancer has been steadily increasing each year in both men and women. Oral squamous cell carcinoma accounts for 80–90% of all oral cancers and is associated with poor prognosis. It is a challenging cancer to treat due to its impact on essential functions such as chewing, speech, and swallowing. The 5-year survival rate for oral cancer patients is just over half, which is significantly lower than other types of cancer. Survival rates drastically decrease in cases with regional or distant metastasis compared to those without metastasis [2].

The global burden of oral cancer is heavily influenced by various risk factors, with tobacco consumption being the most significant contributor. Both smoked and smokeless forms of tobacco are linked to a heightened risk of oral cavity and oropharyngeal cancers [1, 3, 4]. Alcohol consumption is another well-established risk factor, particularly when combined with tobacco use, amplifying the risk through a synergistic effect. In South and Southeast Asia, betel nut chewing, with or without tobacco, plays a critical role, particularly in female populations [1, 3–5]. Recent studies suggest that cultural factors such as the acceptance of smokeless tobacco and betel nut chewing among women may contribute to rising oral cancer incidence in females, especially in parts of Asia. Additionally, lifestyle changes, human papillomavirus (HPV)-related infections, and alcohol use are increasingly reported among female populations, potentially influencing this trend [1, 3–5]. These observations highlight the importance of conducting stratified analyses by sex, as the epidemiological characteristics of oral cancer can vary significantly between men and women across different regions.

In recent years, the role of HPV has gained recognition, especially in the rise of oropharyngeal cancers (oral cancer) [6]. The World Health Organization (WHO) determined that HPV type 16 is implicated in the development of oral cancer [7]. HPV-16 and HPV-18, particularly associated with oral cancer, are frequently implicated in malignancies of the tonsils and base of the tongue [3, 6]. This viral etiology marks a shift from traditional risk factors, as HPV-positive cancers exhibit different epidemiological patterns, such as increased incidence in younger populations and non-smokers [8].

Epidemiological studies conducted in the United States [9], Japan [10], Denmark [11], and Brazil [12] consistently report a shift in oral cancer patterns, with increasing incidence in anatomical sites associated with HPV infections, particularly among women and younger populations. This trend contrasts with the traditionally high prevalence of tobacco- and alcohol-related oral cancers, highlighting a growing concern for HPV-driven malignancies in these regions.

A recent comprehensive analysis of head and neck squamous cell carcinoma epidemiology in South Korea by Jung et al. [13] from 1999 to 2017 revealed contrasting trends. They reported stabilization of HPV-related oropharyngeal cancer incidence after a period of rapid increase, whereas HPV-unrelated oral cavity cancer uniquely showed a continuous increase. This increase was predominantly driven by tongue cancer, exhibiting a particularly steep rise in younger age groups, underscoring the need for updated oral cancer studies.

Analyzing long-term trends in oral cancer is crucial for understanding its epidemiological characteristics and evaluating the effectiveness of prevention and early detection programs. By studying long-term trends, we can identify shifts in the epidemiology of oral cancer, including changes in risk factors, and assess the impact of public health interventions over time. This is essential for identifying emerging patterns and risk groups, which can inform future prevention strategies and targeted interventions. Identifying trends in increased incidence across specific sites or age groups is essential for designing targeted prevention campaigns and implementing precise screening programs. Despite its importance, systematic long-term trend analyses in Korea remain limited.

This study aimed to analyze long-term trends in oral cancer incidence and mortality from 2001 to 2021, assessing site-specific patterns and epidemiological changes, particularly using the latest oral cancer database from Korea to conduct a 20-year analysis that includes the COVID-19 pandemic period. It also aimed to assess the variations in oral cancer incidence across different age groups and sex, with a particular focus on the increasing rates to identify high-risk populations.

## Methods

### Data source

Data from a cohort of patients diagnosed were analyzed using the Cancer Registration Statistics from the Ministry of Health and Welfare from 2001 to 2021 [14]. Korea operates a nationwide, population-based cancer registry system through the Korea Central Cancer Registry (KCCR), established in 1980 under the Ministry of Health and Welfare. The KCCR collects high-quality data on cancer incidence, treatment, and survival, providing essential information for cancer surveillance and public health planning. Data on deaths from malignant neoplasms of the lip, oral cavity, and pharynx were sourced from the cause-of-death statistics provided by Statistics Korea [15]. Cancers included in this study were defined according to the 10th edition of the International Classification of Diseases (ICD-10) [16] as follows: lip (C00), tongue (C01-C02), mouth (C03-C06), salivary glands (CO7-C08), tonsil (C09), oropharynx (C10), nasopharynx (C11), hypopharynx (C12-C13), Other and unspecified sites (C14).

### Ethics statement

This study was approved by the Research Ethics Committee of the Graduate School of Dentistry, Seoul National University (IRB number: S-D20240032).

### Statistical analysis

Based on the data, cancer cases were calculated for overall oral cancer and site-specific subsites, stratified by age group and sex. Incidence and mortality were expressed as age-standardized rates (ASRs) per 100,000 persons, using the direct standardization method. The 2000 Korean resident registration mid-year population was applied as the standard population [15]. Age standardization, which adjusts for differences in age structure, enabled meaningful comparisons of cancer rates across different time periods. The total number of oral cancer cases and their corresponding ASRs were presented over the entire observation period. Subsequently, site-specific oral cancer cases and ASRs were reported in detail by age group and sex.

The total number of oral cancer cases and the age-standardized incidence rates (ASIR) were presented over the entire observation period. Subsequently, the number of cancer cases and ASIRs for all oral cancer cases by subsites were presented, stratified by age group and sex.

Oral cancer incidence and mortality trends from 2001 to 2021 were analyzed using the annual percentage change (APC) with the Joinpoint Regression Program version 5.2.0 [17]. APC represents the yearly rate of change in age-standardized rates within a given time interval. The average annual percent change (AAPC) was also calculated to summarize the overall trend across the entire study period. Although APC and AAPC are distinct measures—APC being calculated for each segment and AAPC representing a weighted average of multiple segments—no significant joinpoints were detected in this study. Therefore, the APC and AAPC were numerically identical and interpreted as the average yearly rate of change over the 20-year period. The joinpoint model evaluates shifts in linear trends across consecutive time intervals. For the trend analysis by specific sites, stratified by age and sex, patients were divided into three age categories: younger than 45 years, 45–64 years, and 65 years or older.

Finally, after calculating the age-standardized mortality rate (ASMR)s for patients diagnosed with oral cancer who had deceased, the trends in ASIRs and ASMRs over the entire period were presented and compared.

Microsoft Excel was primarily used for preliminary data organization and basic tabulation, while R programming language (version 4.4.3) with R Studio (Build 563) were employed to generate graphs and visualizations that effectively illustrate key trends in oral cancer. In R, we used the libraries readxl, dplyr, tidyr, and purrr to import and tidy the Excel sheets into a single long-format data frame of annual age-standardized incidence rates by site and age group. We then leveraged ggplot2’s facet_wrap() to generate a total of 3 figures, each comporised of a 3 × 3 grid of solid annual rate lines overlaid with dashed LOESS smoothing curves, including 95% confidence interval ribbons shaded in gray.

## Results

### Trends in age-standardized incidence rates of overall oropharyngeal cancer

**Fig. 1:**
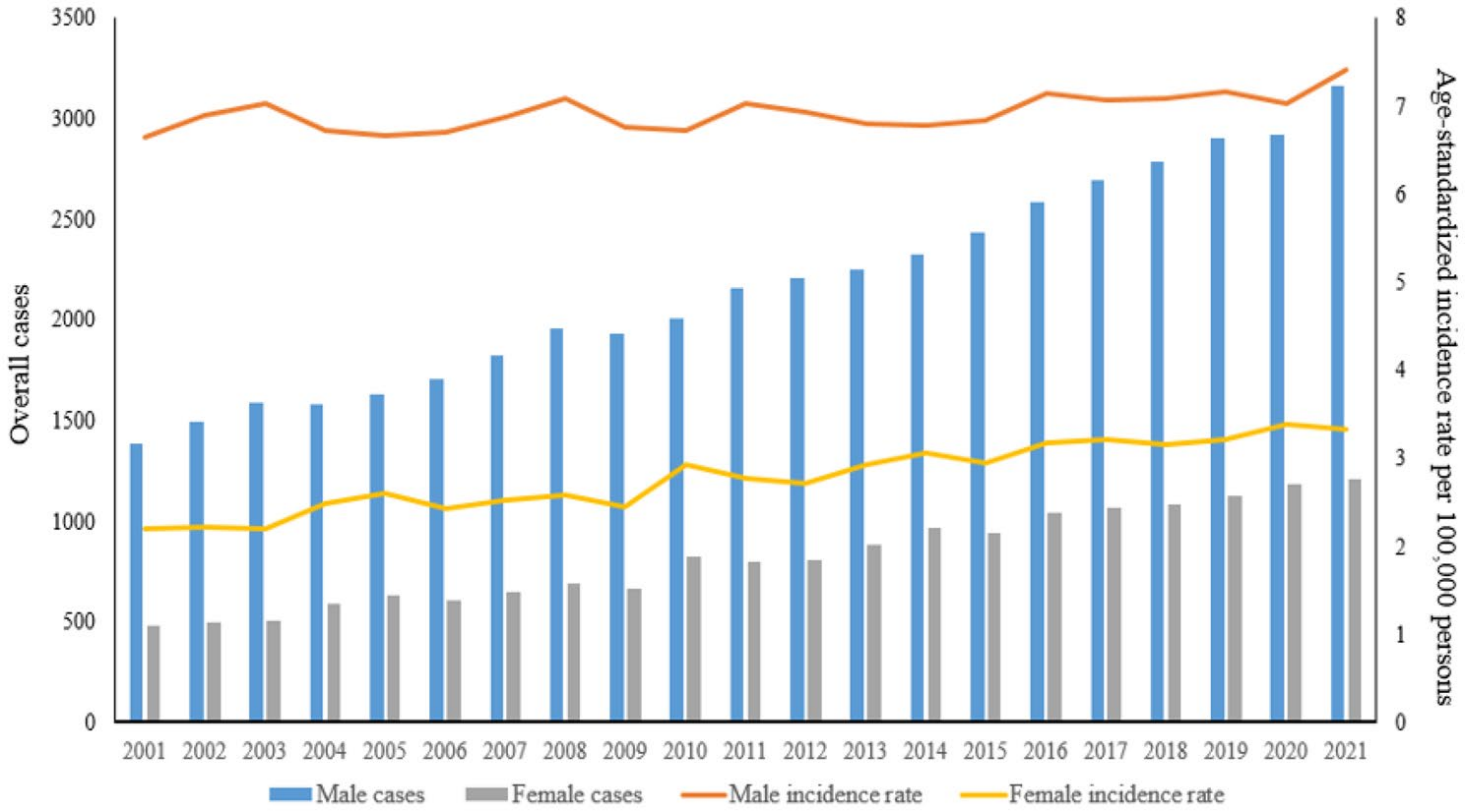
Overall cancer case (C00-C14) distribution and age-standardized incidence rate by sex among South Korea from 2001 to 2021

A total of 4,371 cases of oral cancer were identified, comprising 3,159 cases in males and 1,212 cases in females during the 20-year study period (Fig. 1). Figure 1 presents the estimated ASIRs of oral cancer per 100,000 individuals for both men and women. In 2021, the ASIR for overall oral cancer was estimated at 7.41 per 100,000 men and 3.34 per 100,000 women.

**Fig. 2:**
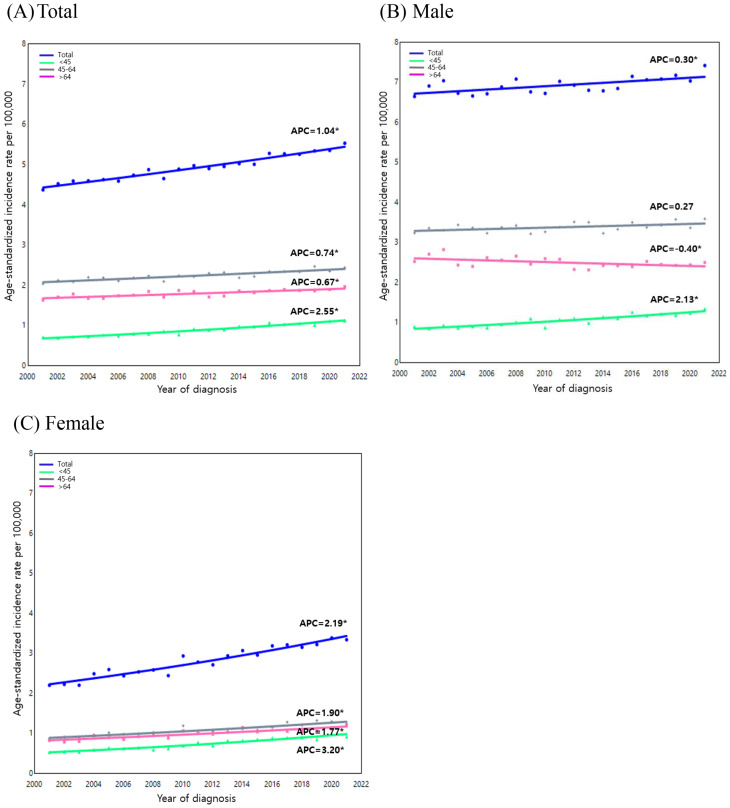
Jointpoint analysis of oral cancer incidence trends (2001–2021) for all cancer sites by age group. (**A**) Total (**B**) Male (**C**) Female. APC, annual percentage change. Solid symbols indicate that the APC is significantly different from zero (*p* < 0.05, two-sided). Trends were analyzed using the Joinpoint Regression Program, version 5.2.0 (National Cancer Institute)

When analyzed by age group and sex, the overall incidence of oral cancer exhibited a substantial increase in females (APC: 2.19, 95% CI: 1.86 to 2.52, *p* < 0.001), while the upward trend in males remained modest (APC: 0.30, 95% CI: 0.08 to 0.53, *p* = 0.0139) (Fig. 2). In males, the incidence showed a slight decrease in those aged 65 years and older (APC: -0.40, 95% CI: -0.78 to -0.02, *p* = 0.040), while the greatest increase was observed in those aged 45 years or younger (APC: 2.13, 95% CI: 1.61 to 2.63, *p* < 0.001). In females, an increasing trend was observed across all age groups, particularly in those aged 45 years or younger (APC: 3.20, 95% CI: 2.72 to 3.66, *p* < 0.001) (Table S1).

### Trends in age-standardized incidence rates of oropharyngeal cancer by subsites

**Fig. 3:**
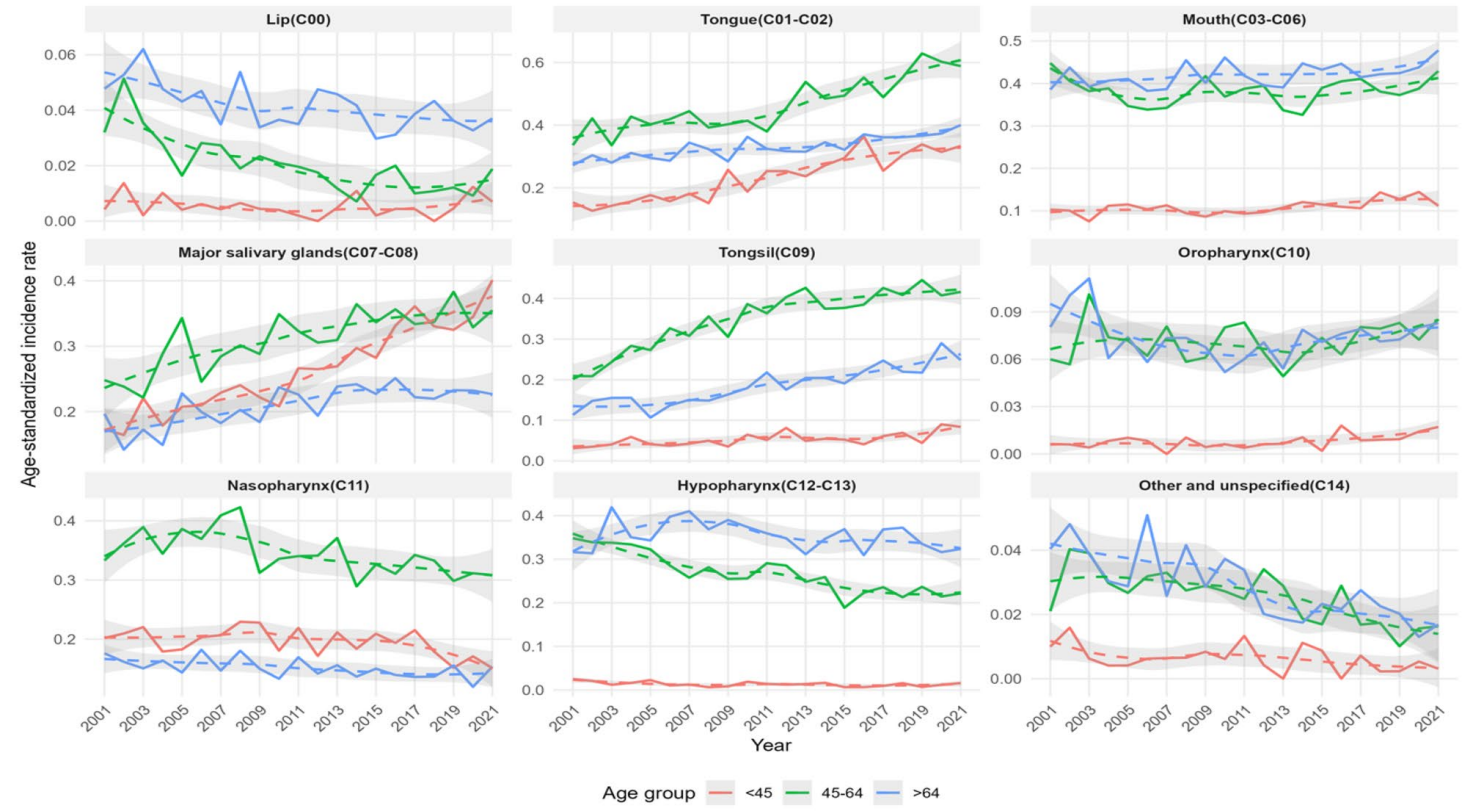
Trends in age-standardized incidence rates and number of cases of oral cancer by subsite and age group among males and females in South Korea from 2001 to 2021

**Fig. 4:**
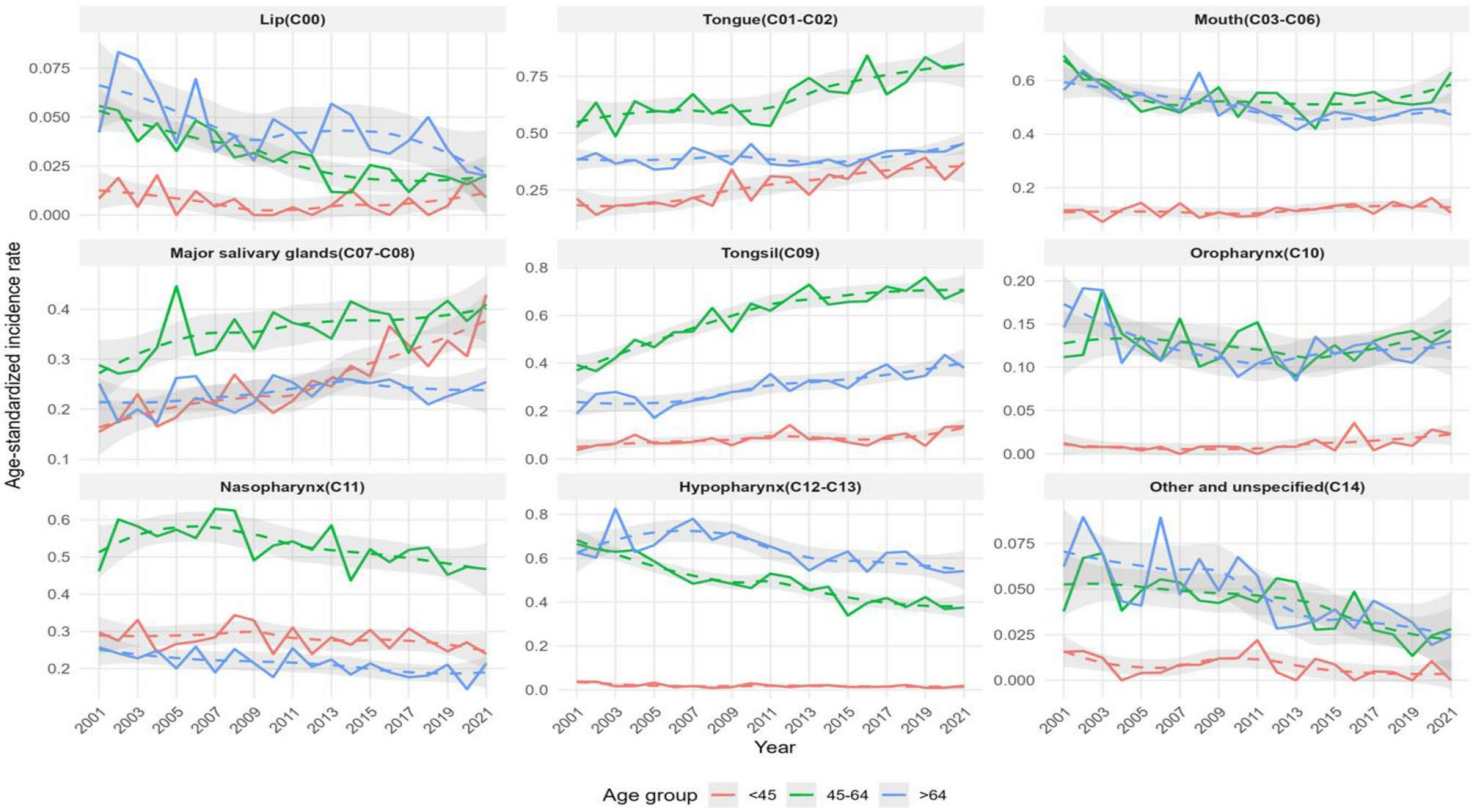
Trends in age-standardized incidence rates and number of cases of oral cancer by subsite and age group among males in South Korea from 2001 to 2021

**Fig. 5:**
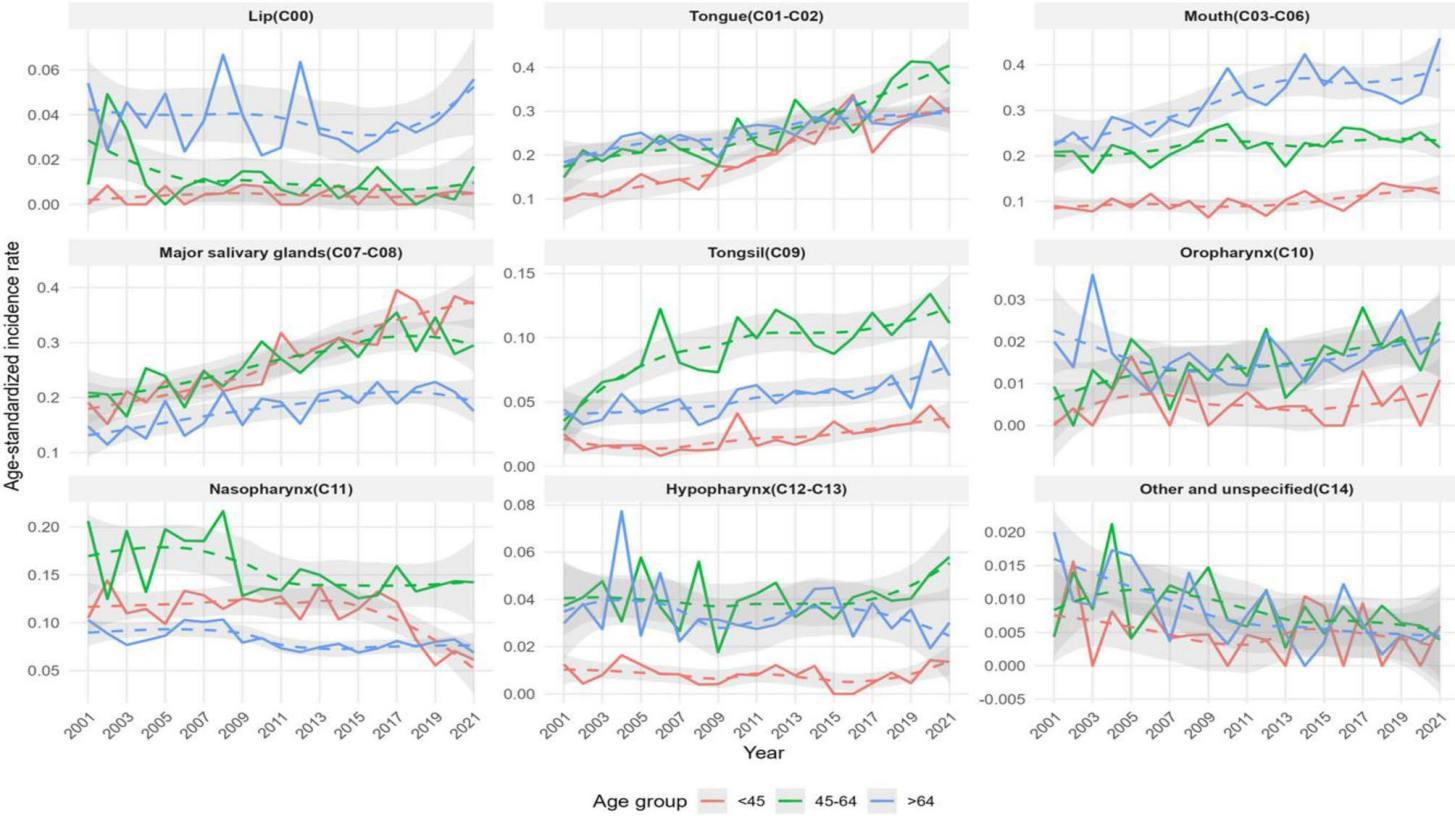
Trends in age-standardized incidence rates and number of cases of oral cancer by subsite and age group among females in South Korea from 2001 to 202

The ASIRs and cancer cases for males and females, categorized by subsites, are presented in Figs. 3, 4, and 5. The overall trends observed in the graphs for the total population and for males and females showed similar patterns.

**Fig. 6:**
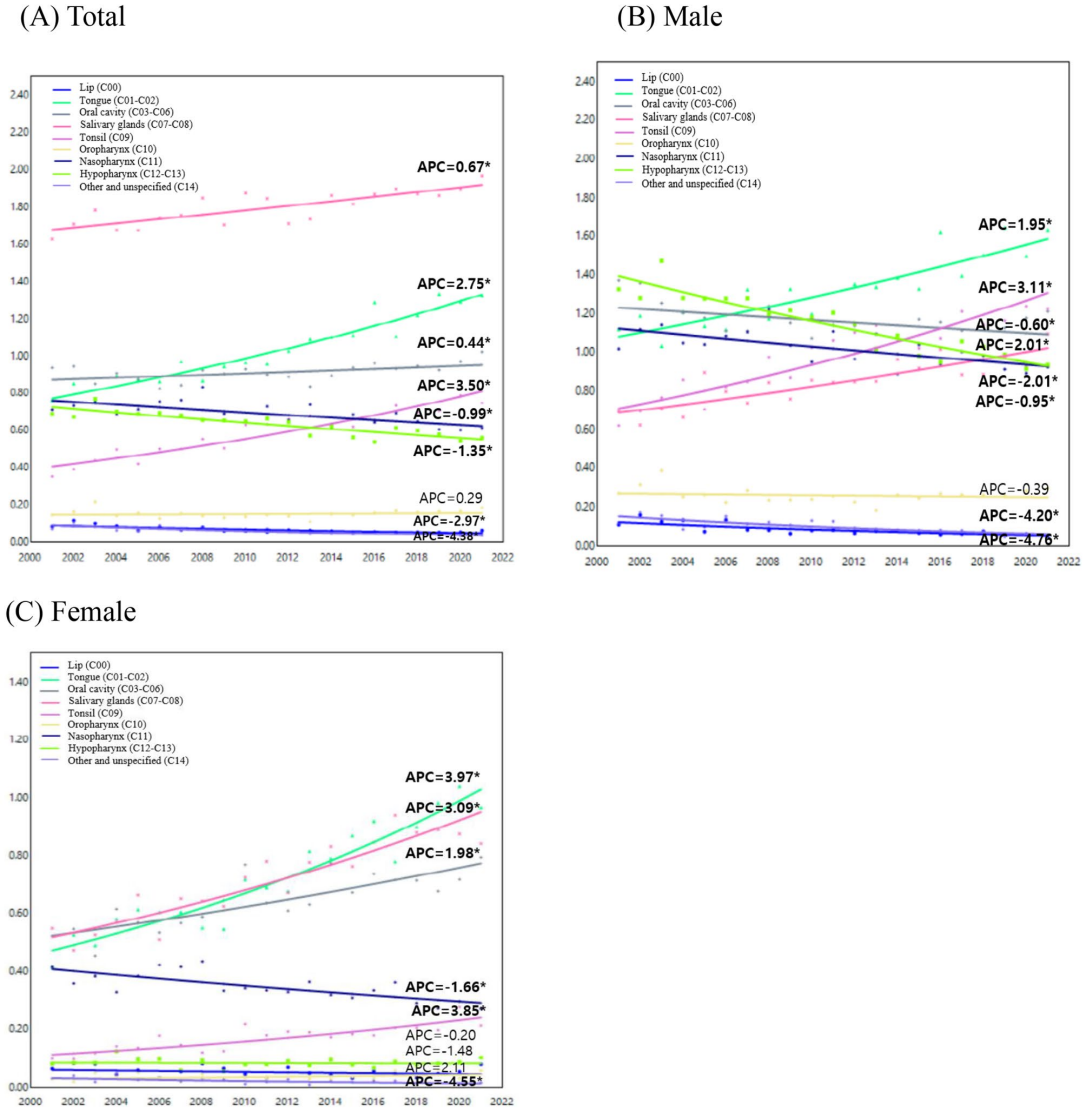
Trends in age-standardized incidence rates of oral cancer by subsite in South Korea from 2001 to 2021. (**A**) Total (**B**) Male (**C**) Female. Solid symbols indicate that the APC is significantly different from zero (*p* < 0.05, two-sided). APC, annual percentage change. Trends were analyzed using the Jointpoint Regression Program, version 5.2.0 (National Cancer Institute)

Figure 6 illustrates the APC, allowing for an analysis of the trends in oral cancer by subsite across different sex. Notably, a more pronounced increase was observed in females over the 20 years. In males, the incidence increased most significantly in the tonsils (APC: 3.11, 95% CI: 2.35 to 3.85, *p* < 0.001), followed by the salivary glands (APC: 2.01, 95% CI: 1.23 to 2.76, *p* < 0.001) and tongue (APC: 1.95, 95% CI: 1.25 to 2.63, *p* < 0.001) (Fig. 6B). In contrast, the greatest increase in females was observed in the tongue (APC: 3.97, 95% CI: 3.18 to 4.72, *p* < 0.001), followed by the tonsils (APC: 3.85, 95%CI: 2.42 to 5.26, *p* < 0.001), salivary glands (APC: 3.09, 95% CI: 2.33 to 3.84, *p* < 0.001), and oral cavity (APC: 1.98, 95% CI: 1.28 to 2.69, *p* < 0.001) (Fig. 6C). It was also observed that both males and females experienced a significant decline in the incidence of cancer in the lip (APC_total_: -2.97, 95% CI: -4.03 to -1.90, *p* < 0.001; APC_male_: -4.20, 95% CI: -5.69 to -2.67, *p* < 0.001; APC_female_: -1.48, 95% CI: -4.08 to 1.11, *p* = 0.259) and other unspecified sites (APC_total_: -4.38, 95% CI: -5.87 to -2.92, *p* < 0.001; APC_male_: -4.76, 95% CI: -6.23 to -3.29, *p* < 0.001; APC_female_: -4.55, 95% CI: -7.26 to -1.85, *p* = 0.0012) (Table S2).

### Annual percentage changes in oropharyngeal cancer incidence rates by subsites according to age groups

**Fig. 7:**
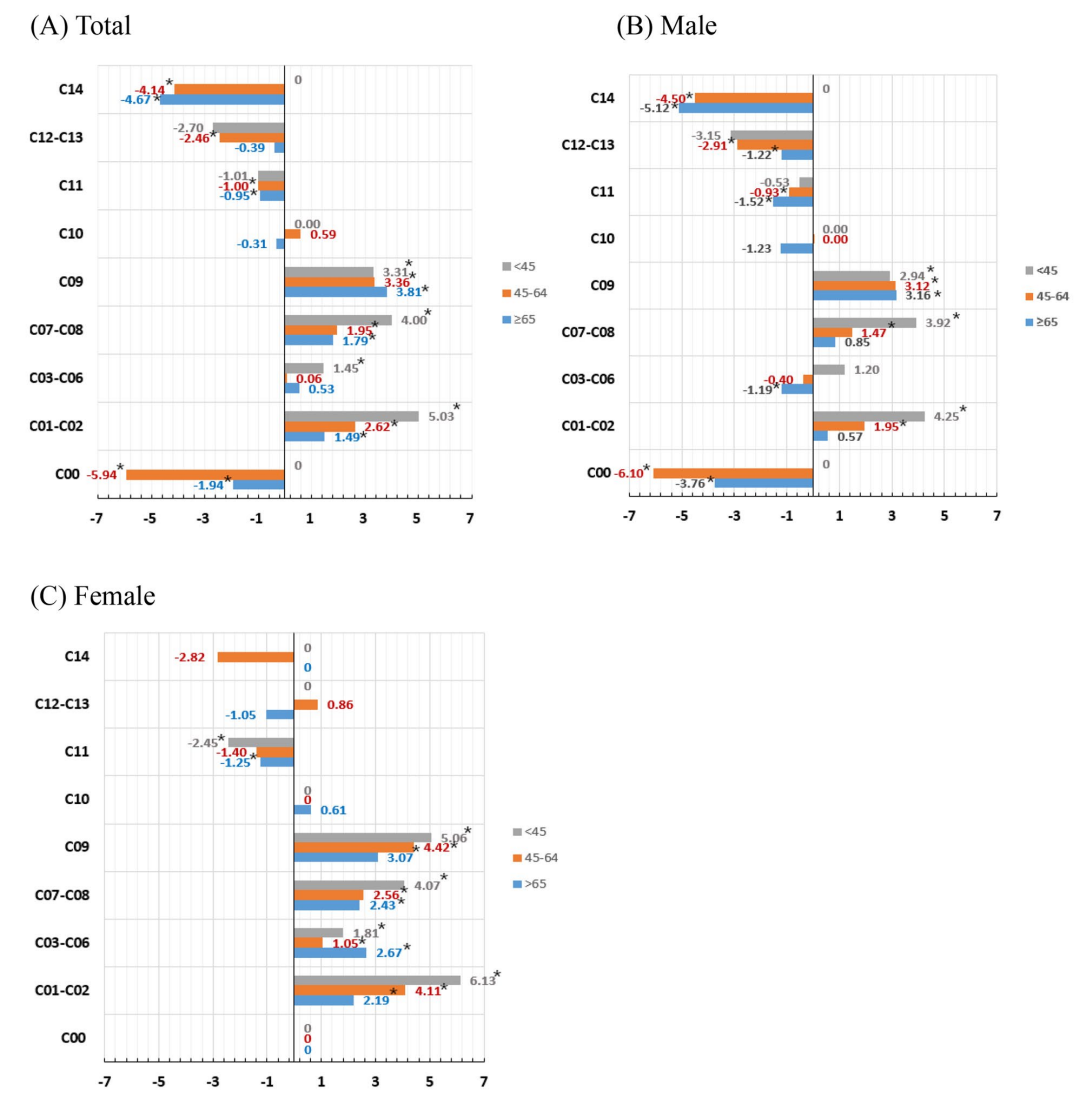
Annual percentage change in incidence rates of oral cancer by site and age group in South Korea from 2001 to 2021. (**A**) Total (**B**) Male (**C**) Female. Solid symbols indicate that the APC is significantly different from zero (*p* < 0.05, two-sided). C00: Lip, C01-C02: Tongue, C03-C06: Mouth, C07-C08: Major salivary glands, C09: Tonsil, C10: Oropharynx, C11: Nasopharynx, C12-C13: Piriform sinus, hypopharynx, C14: Other and unspecified sites of oral cavity and pharynx. Trends were analyzed using the Jointpoint Regression Program, version 5.2.0 (National Cancer Institute)

When analyzed by age group in males, the tongue subsite showed the most significant increase, with an APC of 4.25 (95% CI: 2.32 to 6.13, *p* < 0.001), followed by the salivary glands subsite, which exhibited an APC of 3.92 (95% CI: 2.84 to 5.04, *p* < 0.001) in individuals aged 45 years or younger. Additionally, the tonsil subsite displayed a similar pattern of increase across all age groups. In contrast, in females, the tongue subsite exhibited a substantial increase, with an APC of 6.13 (95% CI: 5.03 to 7.28, *p* < 0.001) in individuals aged 45 years or younger. The tonsil subsite followed with an APC of 5.06 (95%CI: 2.09 to 8.05, *p* < 0.001), while the salivary glands subsite demonstrated an APC of 4.07 (95% CI: 2.87 to 5.24, *p* < 0.001), indicating significant growth in this age group (Fig. 7, Table S3).

### Age-standardized incidence and mortality rates of oropharyngeal cancer in Koreans

**Fig. 8:**
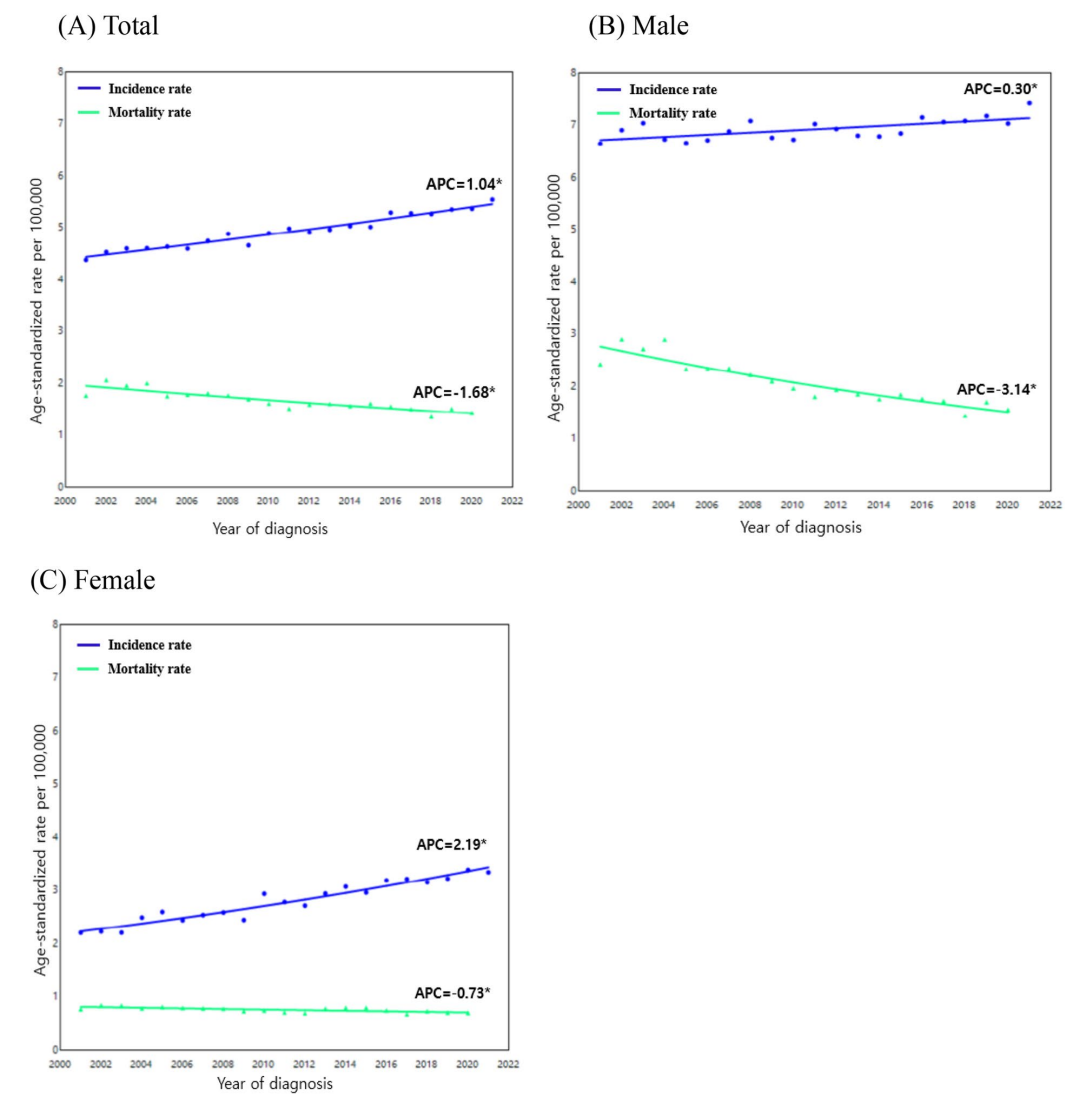
Jointpoint analysis of age-standardized incidence and mortality trends in oral cancer per 100,000 persons in South Korea from 2001 to 2021. (**A**) Total (**B**) Male (**C**) Female. Solid symbols indicate that the APC is significantly different from zero (*p* < 0.05, two-sided). APC, annual percent change. Trends were analyzed using the Jointpoint Regression Program, version 5.2.0 (National Cancer Institute)

Based on the provided figures illustrating the trends in ASIRs of oral cancer for males and females from 2001 to 2021 (Fig. 8). For males, the ASIRs also demonstrated an upward trend, albeit at a slower rate compared to females, with an APC of 0.30 (95% CI: 0.08 to 0.53, *p* = 0.013). This indicates a gradual increase in oral cancer cases among males over the same period. Conversely, the ASMR for males showed a more pronounced decline, with an APC of -3.14 (95% CI: -3.76 to -2.54, *p* < 0.001), indicating a significant reduction in mortality associated with oral cancer among males. The data indicates a significant upward trend in the ASIR for females, with an APC of 2.19 (95% CI: 1.86 to 2.52, *p* < 0.001). This trend highlights a continuous increase in oral cancer cases among the female population over the study period, particularly over the past 20 years. In contrast, the mortality rate for females exhibited a slight decline, with an APC of -0.73 (95% CI: -1.175 to -0.270, *p* = 0.001), suggesting an overall improvement in survival (Table S4).

## Discussion

This study was conducted based on an oral cancer incidence and mortality database from South Korea to identify long-term trends in oral cancer. The findings aim to contribute to the development of effective strategies for the prevention and early detection of oral cancer in the future. From 2001 to 2021, the oral cancer incidence in South Korea was meticulously analyzed by sex, age group, and anatomical sites. In particular, differences in oral cancer incidence by anatomical site between men and women were examined, and the incidence patterns for each anatomical site were analyzed by age group, highlighting the variations.

While the number of oral cancer cases nearly doubled between 2001 and 2021, the age-standardized incidence rate (ASIR) remained relatively stable. This pattern likely reflects the demographic transition in Korea during the study period, particularly the rapid aging of the population. Since the ASIR adjusts for changes in age distribution, increases in case counts among older adults—who are at higher risk for oral cancer—may not have substantially influenced the overall ASIR.

This study’s findings reveal a distinct epidemiological shift in the spectrum of oral cancer disease in Korea over the past two decades. While the overall incidence showed a modest increase in males (APC: 0.30, *p* = 0.0139), females exhibited a significant upward trend (APC: 2.19, *p* < 0.001). Particularly striking was the substantial increase in younger age groups, with the highest rise observed in females aged 45 or younger (APC: 3.20, *p* < 0.001), and a notable increase also seen in males of the same age group (APC: 2.13, *p* < 0.001). Our findings align with those of global trends from the analysis of the GLOBOCAN database collected by the International Agency for Research on Cancer across 185 countries [18], which noted a decline in oral cancer incidence among men while observing an increasing trend in some female populations. Additionally, a recent analysis of Global Burden of Disease study data confirmed an increase in oral cancer incidence among females and individuals aged 15 to 49 [19].

Exploring the possible causes for these shifting patterns, particularly the increase in younger females, is crucial. In some Asian regions, factors such as cultural acceptance of smokeless tobacco and betel nut chewing, along with lifestyle changes, HPV-related infections, and alcohol use, have been suggested as contributors to rising oral cancer incidence in females [1, 3]– [4]. While our study did not have individual-level risk factor data, these broader observations may offer context. It is also important to note that self-reported female smoking rates in surveys [20] may not accurately reflect true prevalence due to conservative societal attitudes, highlighting the need for more rigorous data collection on smoking-related metrics for young women.

The changing spectrum of oral cancer is clearly evident in the subsite-specific trends observed in this study, with a marked decreasing trend in the incidence of lip and other specified sites across the overall population over the past two decades, particularly when analyzed by anatomical site and sex. Notably, there was a significant decline in the incidence of lip cancer, particularly among males (APC: -4.20, *p* < 0.001), a finding consistent with previous Korean and U.S. studies [9, 13]. In contrast, no significant change was observed in female lip cancer incidence (APC: -1.48, *p* = 0.259). The decline among men may be associated with the steady reduction in smoking rates in the Korean male population over the past two decades [20]. Conversely, the absence of a significant decline among women may be partially explained by the limitations of self-reported smoking data and potential underreporting due to social desirability bias [20]. Therefore, more rigorous and objective assessment of smoking-related metrics is essential to support future studies on oral cancer incidence among young women.

The increase in tongue and tonsil cancers is particularly relevant to the changing landscape of oral cancer etiology. These sites are recognized as HPV-associated cancer sites, where specific HPV DNA is frequently detected [8, 21, 22]. HPV-16 and HPV-18 are strongly linked to malignancies of the tonsils and base of the tongue [23]. Notably, HPV-positive cancers often exhibit different epidemiological patterns than those linked to traditional risk factors (smoking and alcohol), including increased incidence in younger populations and non-smokers [8]. Our findings of substantial increases in tongue and tonsil incidence, especially in individuals under 45 years of age, are consistent with reported increases in these sites as corroborated by epidemiological studies conducted in United States [9], Japan [10], Denmark [11], the Brazil [12], Netherlands [24], France [25]. The growing prevalence of oral sexual activities has also been suggested to play a role in this rising incidence [22]. These observations underscore the potential increasing contribution of HPV infection to oral cancer incidence and support the importance of HPV vaccination programs.

The observed increase in salivary gland cancer incidence in this study, with an annual percent change (APC) of 2.01 for men and 3.09 for women, contributes to the changing spectrum of oral cancer. This trend mirrors findings from other studies [10, 26]. For instance, a study by Kawakita et al. [10], which analyzed data from 19 population-based cancer registries in Japan between 1993 and 2015, also identified an increasing trend in salivary gland cancer incidence, reporting an APC of 2.2 for men and 3.1 for women. Both studies indicate a slightly higher increase in incidence among women. Additionally, Del Signore and Megwalu reported an increasing trend (APC of 0.99) using the Surveillance, Epidemiology, and End Results database from the United States between 1973 and 20,093 [26]. In contrast, Aegisdottir et al. [27], using the Icelandic Cancer Registry and Nordic Cancer Registries database, reported no significant change in incidence rates from 1986 to 2015 in those datasets. The exact causes of salivary gland cancer remain unclear. However, potential contributing factors reported in the literature include smoking, alcohol consumption, and radiation exposure [28–31]. While direct exposure to carcinogenic substances, dietary habits, and family history have also been considered [26, 29], the evidence supporting these associations is relatively limited. Thus, the reasons for the increasing trend in salivary gland cancer require further investigation.

Despite the overall increasing trend in oral cancer incidence among Koreans over the past two decades (APC: 1.04), a significant decline in mortality was observed (APC: -1.68), suggesting improved clinical outcomes. This favorable shift may be attributed to advances in diagnostic accuracy, treatment technologies, and heightened public awareness that has encouraged earlier detection—particularly within the framework of the national health insurance system. Notably, male mortality showed a more pronounced decline (APC: -3.14), which may be linked to the steady reduction in smoking rates among Korean men during the same period [20].

In contrast, among women, the incidence of oral cancer has continued to rise, while the decline in mortality has been more modest. This discrepancy could reflect a combination of factors, including possible underreporting of smoking behaviors due to social desirability bias and the increasing contribution of HPV-related cancers, particularly in younger age groups. This multi-faceted issue highlights the need for continued research into the factors affecting oral cancer incidence and mortality.

According to the Korea annual report of the National Cancer Registry, the five-year relative survival rate for oral cancer has shown a continuous increase from 1993 to 2021. Specifically, the survival rate for men rose from 36.7 to 66.6%, an increase of 44.9%. In contrast, while the survival rate for women increased from 59.4 to 77.7%, reflecting a rise of 23.5%, it indicates that the survival rate for men is approximately 1.9 times higher than that for women. In this way, as oral cancer mortality rates decline and the number of survivors continues to rise, it is increasingly important not only to focus on post-treatment surveillance following surgical, radiation, or chemotherapy interventions but also to develop programs aimed at enhancing the quality of life for these patients. A study by Lee et al. [32] demonstrated that comprehensive oral care provided by dental professionals led to improvements in both oral health and quality of life for head and neck cancer patients. To enhance the quality of healthcare services for HNC patients, it is essential to foster communication and collaboration between healthcare providers and dental professionals.

Our study has a few limitations. First, while this study utilized national cancer registry data, it lacked individual-level information on behavioral risk factors such as smoking status, alcohol consumption, and HPV infection, which are known to influence oral cancer development. As such, the inability to control for these confounding variables may limit the interpretation of the observed trends. Second, although age-standardized rates were applied, changes in the underlying population structure and potential misclassification in anatomical subsites reporting may have affected the accuracy of the trend analysis. Third, the absence of HPV vaccination data and its coverage in different age and sex groups poses a challenge in assessing its direct impact on HPV-related oral cancer trends. Finally, cultural and social factors, such as stigma around smoking among women and variations in sexual behavior, were not accounted for, which may result in underestimation or misinterpretation of certain findings. Future research should incorporate more comprehensive data, including behavioral, virological, and sociodemographic factors, to better understand the multifactorial nature of oral cancer incidence and its changing patterns.

It is recommended that, as the incidence of oral cancer continues to rise, it is critical to address this growing burden through a combination of prevention and intervention strategies. These strategies should include the expansion of HPV vaccination programs, early detection initiatives, and public health campaigns aimed at reducing smoking and promoting safe sexual practices. Enhanced diagnostic techniques and personalized treatment plans, along with interdisciplinary collaboration between healthcare providers, will be key in improving outcomes for patients. It is essential to prioritize these efforts in both policy-making and clinical practice to curb the increasing trend of oral cancer and improve survival rates in the future.

## Conclusion

Based on our findings on the trends in oral cancer incidence and mortality, this study highlights the need for continuous monitoring and further research to understand the underlying causes of these patterns. While our study did not directly assess specific risk factors, the observed differences by age, sex, and cancer subsite suggest the importance of exploring potential contributing factors in future studies. These insights may support the development of more targeted public health strategies, including early detection efforts and tailored prevention programs, to reduce the burden of oral cancer.

## Electronic supplementary material

Below is the link to the electronic supplementary material.

Supplementary Material 1

## Data Availability

The data that support the findings of this study are listed in the main manuscript.

## Abbreviations

HPV: Human papillomavirus
APC: Annual percentage change
ICD: International Classification of Diseases
ASR: Age-standardized rates
