# Supplementary material for: An epidemiological trend analysis of oral cancer in Korea from 2001 to 2021
Source: BMC Oral Health. 2025 Jul 1;25:969. doi: 10.1186/s12903-025-06351-1 (PMC12220502; doi:10.1186/s12903-025-06351-1)
Supplement: Supplementary file 1 — Supplementary Material 1 [file 12903_2025_6351_MOESM1_ESM.docx]

| Table S1. Trends in oral cancer incidence by age group and sex, 2001-2021 | | | |
| --- | --- | --- | --- |
|  | APC | 95%CI |  |
| *Total* |  |  |  |
| All ages | 1.04^*^ | 0.90 to 1.17 |  |
| <45 | 2.55^*^ | 2.24 to 2.86 |  |
| 45-64 | 0.74^*^ | 0.52 to 0.95 |  |
| ≥65 | 0.67^*^ | 0.38 to 0.95 |  |
| *Male* |  |  |  |
| All ages | 0.30^*^ | 0.08 to 0.53 |  |
| <45 | 2.13^*^ | 1.61 to 2.63 |  |
| 45-64 | 0.27 | -0.07 to 0.60 |  |
| ≥65 | -0.40^*^ | -0.78 to -0.02 |  |
| *Female* |  |  |  |
| All ages | 2.19^*^ | 1.86 to 2.52 |  |
| <45 | 3.20^*^ | 2.72 to 3.66 |  |
| 45-64 | 1.90^*^ | 1.45 to 2.37 |  |
| ≥65 | 1.80^*^ | 1.03 to 2.48 |  |

APC, annual percentage change

95% CI, 95% confidence interval

*APC is significantly different from zero (two-sided p<0.05)

| Table S2. Trends in oral cancer incidence by subsites and sex, 2001-2021 | | |  | |
| --- | --- | --- | --- | --- |
| Subites | APC | 95%CI | |  |
| *Total* |  |  | |  |
| Lip (C00) | -2.97^*^ | -4.03 to -1.90 | |  |
| Tongue (C01-C02) | 2.75^*^ | 2.36 to 3.17 | |  |
| Mouth (C03-C06) | 0.44^*^ | 0.07 to 0.81 | |  |
| Salivary glands (C07-C08) | 0.67^*^ | 0.38 to 0.95 | |  |
| Tonsil (C09) | 3.50^*^ | 2.61 to 4.36 | |  |
| Oropharynx (C10) | 0.29 | -0.83 to 1.41 | |  |
| Nasopharynx (C11) | -0.99^*^ | -1.68 to -0.34 | |  |
| Hypopharynx (C12-C13) | -1.35^*^ | -1.70 to -1.01 | |  |
| Other and unspecified (C14) | -4.38^*^ | -5.87 to -2.92 | |  |
| *Male* |  |  | |  |
| Lip (C00) | -4.20^*^ | -5.68 to -2.67 | |  |
| Tongue (C01-C02) | 1.95 ^*^ | 1.25 to 2.63 | |  |
| Mouth (C03-C06) | -0.60^*^ | -1.17 to -0.03 | |  |
| Salivary glands (C07-C08) | 2.01^*^ | 1.23 to 2.76 | |  |
| Tonsil (C09) | 3.11^*^ | 2.35 to 3.85 | |  |
| Oropharynx (C10) | -0.39 | -1.57 to 0.82 | |  |
| Nasopharynx (C11) | -0.95^*^ | -1.55 to -0.36 | |  |
| Hypopharynx (C12-C13) | -0.20^*^ | -2.39 to -1.64 | |  |
| Other and unspecified (C14) | -4.76^*^ | -6.23 to -3.29 | |  |
| *Female* |  |  | |  |
| Lip (C00) | -1.48 | -4.08 to 1.11 | |  |
| Tongue (C01-C02) | 3.97^*^ | 3.18 to 4.72 | |  |
| Mouth (C03-C06) | 1.98^*^ | 1.28 to 2.69 | |  |
| Salivary glands (C07-C08) | 3.09^*^ | 2.33 to 3.84 | |  |
| Tonsil (C09) | 3.85^*^ | 2.42 to 5.26 | |  |
| Oropharynx (C10) | 2.11 | -1.50 to 5.77 | |  |
| Nasopharynx (C11) | -1.66^*^ | -2.38 to -0.92 | |  |
| Hypopharynx (C12-C13) | -0.20 | -1.69 to 1.29 | |  |
| Other and unspecified (C14) | -4.55^*^ | -7.26 to -1.85 | |  |

APC, annual percentage change

95% CI, 95% confidence interval

*APC is significantly different from zero (two-sided p<0.05)

| Table S3. Trends in oral cancer incidence by subsites, age group, and sex, 2001-2021 | | | | | |  |  |  |  |
| --- | --- | --- | --- | --- | --- | --- | --- | --- | --- |
|  | Total | |  | Male | | |  | Female | |
| Subsites | APC | 95%CI |  | APC | 95%CI | |  | APC | 95%CI |
| Lip (C00) |  |  |  |  |  | |  |  |  |
| <45 | - | - |  | - | - | |  | - | - |
| 45-64 | -5.94^*^ | -8.32 to -3.56 |  | -6.10^*^ | -8.30 to -3.93 | |  | - | - |
| ≥65 | -1.94^*^ | -3.39 to -0.48 |  | -3.76^*^ | -7.26 to -0.28 | |  | - | - |
| Tongue (C01-C02) |  |  |  |  |  | |  |  |  |
| <45 | 5.03^*^ | 3.75 to 6.29 |  | 4.25^*^ | 2.32 to 6.13 | |  | 6.13^*^ | 5.03 to 7.28 |
| 45-64 | 2.62^*^ | 1.72 to 3.49 |  | 1.95^*^ | 1.13 to 2.77 | |  | 4.11^*^ | 2.36 to 5.80 |
| ≥65 | 1.49^*^ | 0.89 to 2.06 |  | 0.57 | -0.39 to 1.53 | |  | 2.19^*^ | 1.37 to 2.99 |
| Mouth (C03-C06) |  |  |  |  |  | |  |  |  |
| <45 | 1.45^*^ | 0.20 to 2.68 |  | 1.20 | -0.85 to 3.21 | |  | 1.81^*^ | 0.33 to 3.28 |
| 45-64 | 0.06 | -0.62 to 0.73 |  | -0.4 | -1.30 to 0.48 | |  | 1.05^*^ | 0.13 to 1.96 |
| ≥65 | 0.53 | -0.01 to 1.06 |  | -1.19^*^ | -1.91 to -0.49 | |  | 2.67^*^ | 1.54 to 3.78 |
| Salivary glands (C07-C08) |  |  |  |  |  | |  |  |  |
| <45 | 4.00^*^ | 3.29 to 4.74 |  | 3.92^*^ | 2.85 to 5.04 | |  | 4.07^*^ | 2.87 to 5.24 |
| 45-64 | 1.95^*^ | 1.07 to 2.81 |  | 1.47^*^ | 0.50 to 2.49 | |  | 2.56^*^ | 1.51 to 3.60 |
| ≥65 | 1.79^*^ | 0.81 to 2.77 |  | 0.85 | -0.43 to 2.14 | |  | 2.43^*^ | 1.09 to 3.74 |
| Tonsil (C09) |  |  |  |  |  | |  |  |  |
| <45 | 3.31^*^ | 1.53 to 5.17 |  | 2.94^*^ | 0.69 to 5.22 | |  | 5.06^*^ | 2.09 to 8.05 |
| 45-64 | 3.36^*^ | 2.40 to 4.33 |  | 3.12^*^ | 2.36 to 3.91 | |  | 4.42^*^ | 2.11 to 6.71 |
| ≥65 | 3.81^*^ | 2.73 to 4.88 |  | 3.16^*^ | 1.88 to 4.40 | |  | 3.07^*^ | 1.34 to 4.80 |
| Oropharynx (C10) |  |  |  |  |  | |  |  |  |
| <45 | - | - |  | - | - | |  | - | - |
| 45-64 | 0.59 | -1.01 to 2.17 |  | 0.02 | -1.64 to 1.65 | |  | - | - |
| ≥65 | -0.31 | -1.71 to 1.10 |  | -1.23 | -2.96 to 0.46 | |  | 0.61 | -2.36 to 3.62 |
| Nasopharynx (C11) |  |  |  |  |  | |  |  |  |
| <45 | -1.01 | -2.32 to 0.26 |  | -0.53 | -1.56 to 0.46 | |  | -2.45^*^ | -4.18 to -0.72 |
| 45-64 | -1.00^*^ | -1.73 to -0.29 |  | -0.93^*^ | -1.65 to -0.21 | |  | -1.40 | -2.93 to 0.13 |
| ≥65 | -0.95^*^ | -1.77 to -0.13 |  | -1.52^*^ | -2.67 to -0.39 | |  | -1.25^*^ | -2.36 to -0.15 |
| Hypopharynx (C12-C13) |  |  |  |  |  | |  |  |  |
| <45 | -2.70 | -6.54 to 1.25 |  | -3.15 | -6.86 to 0.71 | |  | - | - |
| 45-64 | -2.46^*^ | -3.17 to -1.77 |  | -2.91^*^ | -3.63 to -2.21 | |  | 0.86 | -1.57 to 3.25 |
| ≥65 | -0.39 | -1.29 to 0.49 |  | -1.22^*^ | -2.19 to -0.28 | |  | -1.05 | -3.72 to 1.72 |
| Other and unspecified (C14) | |  |  |  |  | |  |  |  |
| <45 | - | - |  | - | - | |  | - | - |
| 45-64 | -4.14^*^ | -5.94 to -2.32 |  | -4.50^*^ | -6.67 to -2.30 | |  | -2.82 | -6.88 to 1.32 |
| ≥65 | -4.67^*^ | -7.38 to -1.99 |  | -5.12^*^ | -7.94 to -2.32 | |  | - | - |

APC, annual percentage change

95% CI, 95% confidence interval

*APC is significantly different from zero (two-sided p<0.05)

| Table S4. Trends in oral cancer incidence and mortality by sex, 2001-2021 | | |
| --- | --- | --- |
|  | APC | 95%CI |
| *Total* |  |  |
| Incidence | 1.04^*^ | 0.90 to 1.17 |
| Mortality | -1.68^*^ | -2.09 to -1.27 |
| *Male* |  |  |
| Incidence | 0.30^*^ | 0.07 to 0.53 |
| Mortality | -3.14^*^ | -3.76 to -2.54 |
| *Female* |  |  |
| Incidence | 2.19^*^ | 1.86 to 2.52 |
| Mortality | -0.73^*^ | -1.17 to -0.27 |

APC, annual percentage change

95% CI, 95% confidence interval

*APC is significantly different from zero (two-sided p<0.05)
